# Fluridone stimulates in vitro seed germination of a rare hardy terrestrial orchid (*Platanthera leucophaea*)

**DOI:** 10.1186/s40529-025-00484-w

**Published:** 2025-10-30

**Authors:** Rachel E. Helmich, Lawrence W. Zettler, Caleb J. Dvorak, Susanne DiSalvo

**Affiliations:** 1 Department of Biological Sciences, Southern Illinois University Edwardsville, 1 Hairpin Dr., Edwardsville, IL 62650 USA; 2 Missouri Botanical Garden, 4344 Shaw Blvd., St. Louis, MO 63110 USA; 3 Present Address: Donald Danforth Plant Science Center, 975 North Warson Road, St. Louis, MO 63132 USA; 4 Department of Biology, Illinois College, 1101 West College Avenue, Jacksonville, IL 62650 USA; 5 Present Address: Flask Factory, 50107 Flintrock Dr., Georgetown, TX 78626 USA

**Keywords:** Abscisic acid (ABA), Phytohormones, Conservation, Micropropagation, Herbicide

## Abstract

**Background:**

Seeds of temperate terrestrial (hardy) orchids are considered more difficult to germinate compared to their tropical epiphytic counterparts, presumably because they have higher levels of abscisic acid (ABA) in their seed coats which prevents seeds from germinating prematurely during winter dormancy. In nature, ABA is gradually broken down (stripped) by natural weathering, triggering germination. This process can be shortened artificially, however, by using chemical bleaching agents and cold-moist stratification with mixed results. In this study, we explored the use of fluridoneto break seed dormancy in a hardy orchid native to North America, *Platanthera leucophaea* (Nutt.) Lindl. This organic compound (IUPAC name: 1-methyl-3-phenyl-5-[3-(trifluoromethyl) phenyl] pyridin-4(1*H*)-one) is a commercial herbicide that inhibits ABA biosynthesis. We added fluridone directly to agar media prior to seed sowing in vitro. Both symbiotic and asymbiotic germination techniques were applied that involved two different agar media, with and without added fluridone. Symbiotic germination was carried out using standard oatmeal agar inoculated with a mycorrhizal fungus (*Ceratobasidium*), whereas asymbiotic treatments utilized P723 agar medium.

**Results:**

Seedling development within some of the replicate plates progressed to Stage 3 in all treatments, but development was marked in all asymbiotic plates containing fluridone leading to leaf elongation, 385 days after sowing.

**Conclusions:**

As an herbicide, fluridone’s use as a media additive to propagate a rare photosynthetic orchid seems counterintuitive, but its use in vitro to stimulate seedling development has the potential to benefit conservation efforts for this and possibly other hardy orchid species.

**Supplementary Information:**

The online version contains supplementary material available at 10.1186/s40529-025-00484-w.

## Background

The commercially applied aquatic herbicide, fluridone (IUPAC name: 1-methyl-3-phenyl-5-[3-(trifluoromethyl) phenyl] pyridin-4(1*H*)-one), is an organic compound (McCowen et al. 1979) that interferes with phytoene desaturase (PDS) within the carotenoid biosynthesis pathway leading to the breakdown of chlorophyll (Laje et al. 2019). Upon exposure, plants lose their green pigmentation and soon become vulnerable to photobleaching, leading to death. Because this process inhibits photosynthesis, fluridone also blocks the biosynthesis of abscisic acid (= ABA) (Stetsenko et al. 2015). As a phytohormone, ABA is present throughout a plant’s life and performs a variety of roles. For instance, ABA levels protect plants from heat, drought stress, and damage from cold temperatures (Chen and Gusta 1983). ABA is also thought to prevent seeds from germinating prematurely by inducing dormancy until this phytohormone is gradually broken down (stripped) by natural weathering and other factors such as smoke exposure (Kamran et al. 2013). As ABA wears off, environmental cues activate the embryo, triggering germination, seedling growth and development (Brown and Smith 2000; Kucera et al. 2005; Skubacz and Daszkowska-Golec 2017; Haider et al. 2018; Mino et al. 2021). Imbibition, cold stratification, and chemical/physical scarring are common methods used to break dormancy by triggering ABA catabolism; however, some species fail to break dormancy under such methods and in the case of imbibition, ABA synthesis rather than catabolism has been favored (Kermode 2005).

Seeds of many temperate terrestrial orchids are considered more difficult to germinate compared to their tropical epiphytic counterparts, presumably because they have higher levels of ABA in their seed coats (Kinderen 1987; Lee et al. 2007, 2015). As a result, researchers subject seeds to prolonged cold pretreatments (stratification) to break ABA-induced dormancy prior to sowing, as exemplified by the prairie fringed orchids of North America (Zettler et al. 2001; Treher et al. 2015). Considering that many regions of the globe are projected to experience significant weather extremes this century imposed by climate change, understanding ABA’s importance on these physiological mechanisms (e.g., seed germination) has added meaning for orchids and other plants faced with extinction.

For conservation purposes, propagating orchids from seed derived from cross pollination is preferable to cloning because this practice maintains genetic variability bequeathed in natural populations (Jersáková et al. 2006; Bazzicalupo et al. 2025). Sowing seeds in vitro on agar media containing sugars and other additives (= asymbiotic germination) is a time-tested practice dating back to groundbreaking work by Knudson (1922), especially for tropical epiphytic species. This protocol has allowed orchids to be produced en masse on a scale previously impossible, completely changing orchid commercialization in the process (White 1927). For temperate terrestrial orchids, however, asymbiotic germination has been met with mixed results, in part because their seeds have built-in dormancy mechanisms (e.g., higher ABA levels) that must be overcome (Rasmussen 1995). For this reason, other techniques have been employed to propagate the more fastidious species from seed including the use of mycorrhizal fungi to facilitate in vitro seed germination (Rasmussen 1995; Swarts and Dixon 2017).

**Fig. 1:**
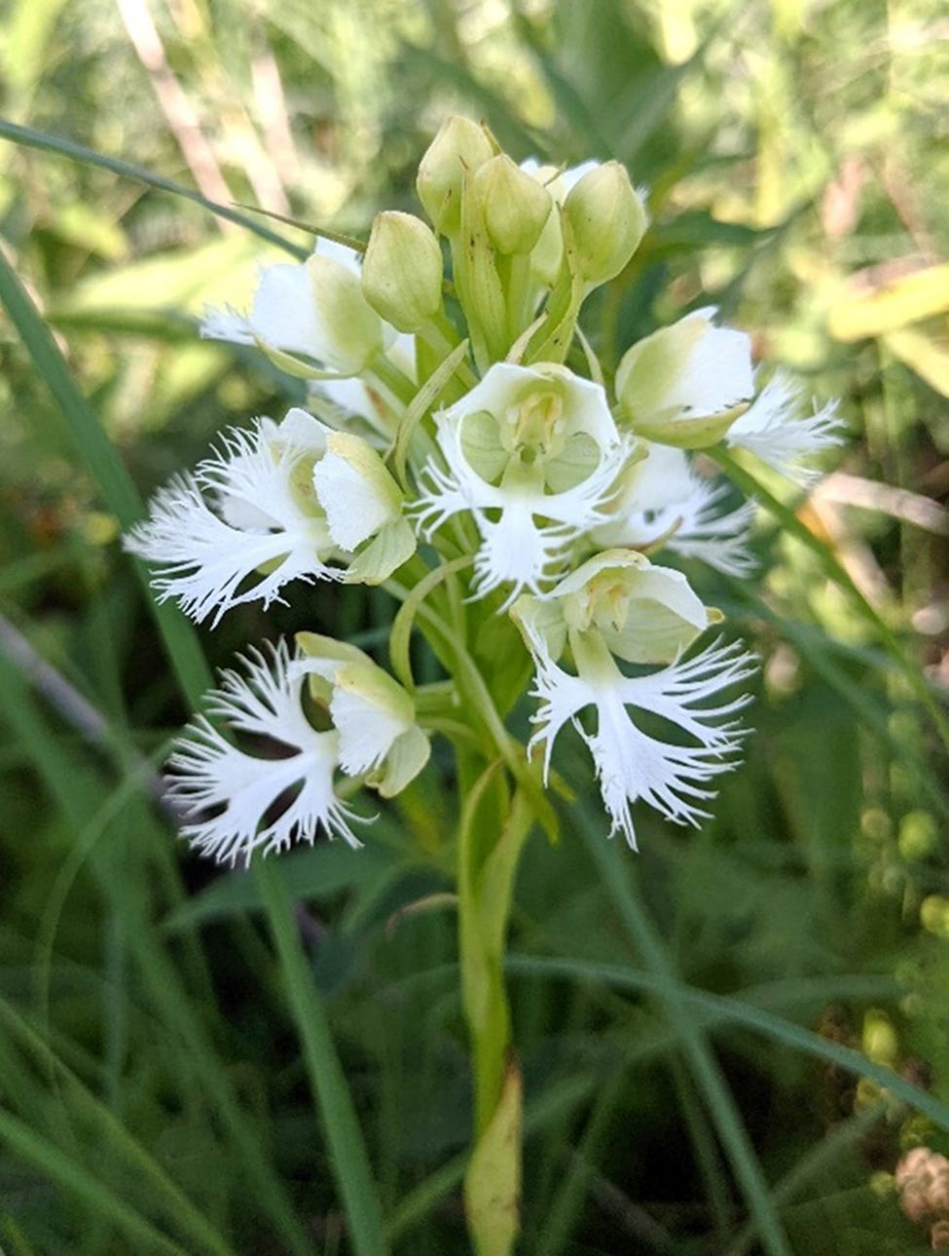
*Platanthera leucophaea* (Nutt.) Lindl. shown in flower at a population in Missouri where seeds used in this study were later collected

One such species that has benefitted from both cold-stratification and symbiotic germination is the eastern prairie fringed orchid of North America, *Platanthera leucophaea* (Nutt.) Lindl., a U.S. Federally threatened species (Fig. 1). As a ‘specialist species’ (Swarts and Dixon 2009), *P. leucophaea* relies exclusively on hawk moths (Sphingidae) for cross pollination (Pollack, 2009), and mycorrhizal fungi (*Ceratobasidium* D.P. Rogers) present in soil for seed germination in situ (Zettler and Piskin 2011; Thixton et al. 2020). Although this orchid has been successfully propagated with fungi to the leaf-bearing stage in vitro by applying cold stratification before and after germination, only a small percentage (< 4%) of seeds with embryos developed into seedlings suitable for reintroduction (Zettler et al. 2005). Clearly, more effective germination protocols aimed at conserving *P. leucophaea*, and other temperate species threatened with extinction need to be developed which serves as the impetus of our study. In particular, we wanted to revisit ABA’s inhibitory role on early seed germination processes in this species, namely, for the purposes of chemically overcoming seed dormancy barriers.

To our knowledge, no studies have explored ABA’s role in hindering seed germination of terrestrial orchids native to North America such as *P. leucophaea*. However, Lee et al. (2007, 2015) experimented with ABA concentrations on seed germination and development of two temperate terrestrials native to Asia, *Calanthe tricarinata* and *Cypripedium formosanum*. In particular, both papers revealed that fluridone – the known ABA biosynthesis inhibitor – may hold considerable promise at stripping ABA’s inhibitory effects. Accordingly, this concept served as the basis of the present study, namely, to compare seed germination and seedling development of *P. leucophaea* with and without fluridone added to agar media. Although our study did not directly test ABA levels, the action of this compound’s role on inhibiting this phytohormone is well established (Gamble and Mullet 1986; Yoshioka et al. 1998; Lee et al. 2015).

## Methods

### Seed collection and storage

Mature seeds of *Platanthera leucophaea* (Nutt.) Lindl., derived from natural pollination, were collected under permit from 1 to 2 capsules on nine separate plants from an undisclosed population in Grundy County, Missouri, USA on 9 August 2021. This population, harboring ca.15 flowering individuals that year, consisted of mesic tallgrass (upland) prairie habitat on loamy soil consistent with other sites in the southwestern portion of its range subject to fire management (Bell et al. 2021). The primary vascular plant associates consisted of *Helianthus grosseserratus* M. Martens, *Lysimachia ciliata* L., *Solidago altissima* L., *Agrimonia pubescens* Wallr., *Carex trichocarpa* Muhl. *ex* Willd., and *Parthenium integrifolium* L. Robust capsules on the verge of dehiscence were selected from each inflorescence, carefully detached to maintain capsule integrity, placed into separate containers, and promptly transported to the laboratory for processing (within 24 h). Upon arrival, the capsules were thoroughly dried over Drierite™ desiccant (CaS0_4_; W. A. Hammond Co.) during the course of one week and removed from capsules following the methods described in Zettler (1997). Seeds were inspected visually to ensure that they were mature and contained robust embryos, evidenced by the testa’s dark brown coloration and rounded embryo shape, respectively, matching published descriptions for this species (Collier et al. 2023). Tetrazolium (TZ) tests were performed in a non-rigorous manner, with noted observations of seed viability when seeds were subjected to longer bleach times as a technique to break down the testa and allow the TZ to access the embryo. After drying, seeds intended for use in this study remained in the micropropagation laboratory for storage at 4–6 °C in a flask with silica beads to maintain low (< 5%) humidity for approx. 19 week until use, while the larger seed stocks were deposited by maternal line in the Missouri Botanical Garden’s Seed Bank. Prior to sowing, a portion of seed from each maternal line was removed from cold storage and pooled into roughly equal quantities for the seed germination experiments that ensued.

### Fungal isolation, identification and storage

To recover and utilize potential mycorrhizal fungi for the purposes of in vitro symbiotic germination, lateral roots of *P. leucophaea* were obtained from mature plants at the same population that provided seed. Root selection, field collection and fungal isolation followed protocols described by Zettler and Corey (2018). Roots were carefully excavated by hand to a depth of ca. 20 cm. which exposed lateral roots marked by orange-yellow patches indicative of regions that harbored living fungal pelotons. One lateral root was detached per plant and the remainder of the root system was left intact, with considerable care taken to return the donor orchid to soil. Roots were promptly returned to the laboratory and placed in refrigeration (4° C) for up to one week before fungal isolation.

Prior to fungal isolation, roots were gently rinsed under running tap water to remove soil debris and surface sterilized for 1 min. in a 5% solution of calcium hypochlorite Ca(ClO)_2_ for 1 min. followed by two 1 min. sterile DI water rinses. Each root was then cut into 1 cm segments, and each segment was transferred to a separate 9 cm diam. Petri dish containing 5 mL of sterile water. The segment was then macerated using a sterile scalpel within the sterile water resulting in fungal pelotons embedded in the cortical region to disperse. Warm molten Fungal Isolation Medium (FIM; Clements et al. 1986) containing an antibiotic (streptomycin sulfate, PhytoTech Labs^®^, product ID: S739) was poured into the dish, gently swirled, and the agar was allowed to cool. After 24–48 h, plates were inspected for actively growing hyphae emerging from pelotons using a dissection microscope. Fungi were isolated in pure culture from hyphal tips excised by use of a sterile scalpel. Each hyphal tip was then transferred to Potato Dextrose Agar (PDA; Difco™, Becton-Dickinson and Co., Sparks, MD, USA), and the plate was immediately sealed using plastic wrap (Packing Supplies By Mail) to retain moisture and restrict potential contaminants from gaining entry. After 1–2 weeks, fungal colonies on PDA were visually inspected for cultural characteristics matching published descriptions for orchid mycorrhizal fungi, namely genera assignable to the form-genus *Rhizoctonia* (Currah et al. 1997). One particular isolate, provisionally identified as a *Ceratobasidium* species, was retained given that members of this genus typically form mycorrhizal associations with *P. leucophaea* (Thixton et al. 2020). This culture, referred herein as Pleuc1.1, was then identified further using molecular techniques.

For molecular identification, ca. 1 cm^3^ of culture Pleuc1.1 was grown in Potato Dextrose Broth (PDB) over the span of 2–3 weeks under continuous agitation via an orbital shaker. After this time, 1 ml was harvested for DNA extraction using the quick-DNA Fungal/Bacterial Miniprep kit (Zymo Research Corporation, Irvine, CA, USA). To PCR amplify the internal transcribed spacer (ITS) ribosomal DNA region for identification, a PCR reaction using two forward primers, ITS1OF.2 (AACTTGGTCATTTAGAGGAAGT) and ITS1OF.1 (AACTCGGCCATTTAGAGGAAGT), in combination with the reverse primer, ITS4OFrev (GTTACTAGGGGAATCCTTGTT) was performed as described in Taylor and McCormick (2008). The PCR product was purified using Exo-sap subjected to Sanger sequencing (Eurofins Genomics). The resulting sequence was queried against the NCBI database using the BLAST algorithm to identify sequences with the highest similarity and has been deposited in GenBank under the accession number PQ140502.

### Media selection and preparation

To fully assess seed germination and seedling development in this species, both symbiotic and asymbiotic germination techniques were applied that involved different agar media. For the former, an oat-based medium (OMA; Dixon 1987) was utilized because of its effectiveness at germinating orchid seeds in vitro using *Ceratobasidum* in previous studies involving *P. leucophaea* (e.g., Zettler et al. 2001). OMA (2.5 g rolled oats/9.0 g agar/1L water) differs from asymbiotic media in that it lacks simple sugars which would otherwise result in rapid overgrowth of the fungus obscuring the seeds preventing visual germination assessment. For asymbiotic germination, the standard medium we used (referred herein as ASYM) consisted of P723 (16.37 g/L; PhytoTech Labs^®^) with added potato flour (6.5 g/L; Bob’s Red Mill^®^), sucrose (10.0 g/L), and agar (5.0 g/L PhytoTech Labs, product ID: A1000). P723 is a complete medium that contains many different ingredients OMA lacks, such as ammonium nitrate, activated charcoal, peptone, and sucrose, just to name a few (full component list is published online and easily accessible). OMA is considered a standard medium used in symbiotic germination because the oats (starch) feed the fungus more gradually, which these fungi are accustomed to in situ given their saprotrophic mode of nutrition. OMA feeds/targets the growth of the fungus so that its hyphae can grow outward physically contacting the seeds, then enter the embryo where the carbon from the fungus is then transferred to the embryo via mycotrophy. By comparison, P723 targets the embryo directly because the sucrose serves as the primary carbon source, not the fungus. Fluridone (15.0 mg/L; Millapore Sigma, product ID: 45511) was added to both OMA and P723 to assess its role in symbiotic and asymbiotic germination experiments and both treatments were labelled as OMA + F and ASYM + F, respectively. Nitrile gloves, long sleeves, safety glasses, and an N95 were worn when working with the pure fluridone powder, as recommended in accordance with the manufacturer’s recommendations, while proper disposal of fluridone should occur in biohazard waste for incineration at an approved waste disposal plant to prevent environmental contamination (Millipore Sigma’s SDS sheet). Media lacking fluridone was prepared, serving as controls. Prior to autoclaving, the acidity of the media was adjusted to a range of pH 5.6–5.8. To ensure the chemical integrity of fluridone in the preparation of sterile media, a stock solution of this compound was added to media in the fluridone treatments after autoclaving once the temperature dropped to 50° C. This was formulated by adding 75 mg of fluridone powder (Millapore Sigma., product ID: 45511) dissolved in 20 mL of 95% laboratory grade ethanol (Carolina Biological, product ID: 861283) rendering a 3.75 mg/mL fluridone stock solution. Following sterilization, the media was poured into sterile 100 mm x 15 mm Petri plates (Cell Treat^®^) within a sterile laminar flow hood and allowed to cool until the agar solidified. To conserve moisture and restrict contaminating microbes, the plates were wrapped tightly using plastic wrap (Packaging SuppliesByMail, SKU 50001-1).

### Seed surface-sterilization

Prior to sowing, seeds were removed from cold storage and chemically scarified. This process involved placing seeds into water absorbent packets (micro-filters; AeroPress^®^, 2.5” diam.) that allowed for chemical exposure while retaining seeds. Approximately 50–300 seeds were added to each packet, and each packet was then secured using standard staples. A total of 160 packets were prepared that were split into 5 different groups representing different seed surface-sterilization times (10, 30, 60, 90, and 120 min; 32 packets per designated time). The surface-sterilized solution consisted of 10% household bleach (VP Specialty Products, Inc, Sunbrite Ultra Bleach – 6.0% NaOCl) + 1 drop/100mL polysorbate 20 (Florida Laboratories, Inc, Polysorbate 20 Food Grade Kosher) + RO water. The packets were agitated at 200 rpm on an orbital shaker for their designated time duration, then moved to flasks containing sterile RO water. Before sowing, packets were further subdivided (*n* = 8) for seed sowing onto 4 different media: OMA, OMA_F, ASYM, and ASYM_F.

### Seed sowing

Seed sowing consisted of reopening each packet under a sterile hood and gently smearing seeds onto the surface of the medium assisted by a sterile forceps. All plates used in symbiotic germination trials (both OMA treatments) were inoculated with *Ceratobasidum* fungus Pleuc1.1 by adding a 1 cm^3^ block of inoculum from the actively growing margins of the original colony. All plates were then individually sealed using plastic wrap and incubated at ambient temperature (20^o^ C) in 24-hr dark photoperiod for 1 week to allow the fungus in the symbiotic plates to proliferate, ensuring that the hyphae made physical contact with the seeds. Plates in all treatments were then incubated in total darkness at 4–6 °C for 90 days for the purposes of cold-moist stratification required for germination in this species (Zettler et al. 2001). After 90 days, the plates were maintained at a 24-hr dark photoperiod at 18–21 °C (nighttime) and 22–26.5 °C (daytime) for 295 days, with data recorded at 385 days after sowing.

### Data collection and analysis

**Fig. 2:**
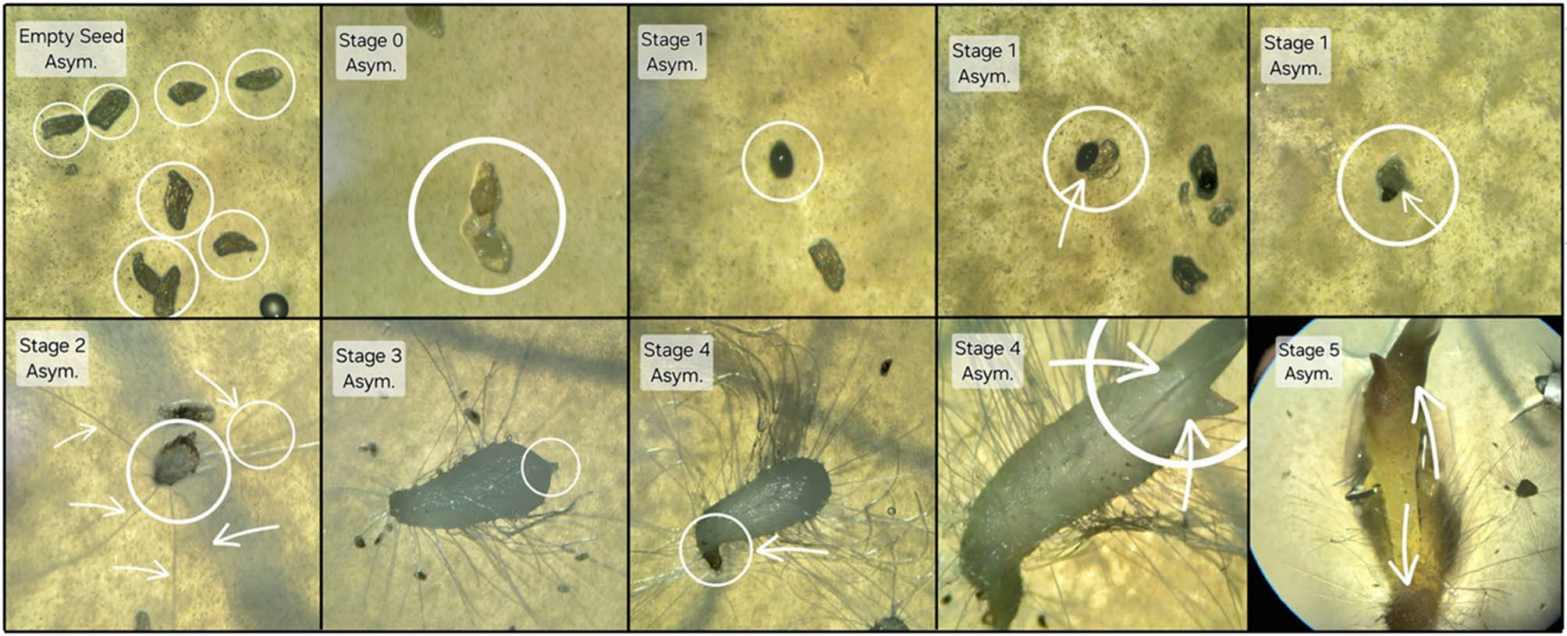
Seed germination and growth stages during *Platanthera leucophaea* development. All pictures taken from asymbiotic treatments. Empty Seed = absent or undeveloped embryos; Stage 0 = embryo present, may be swollen, but testa intact; Stage 1 = no testa present around embryo or testa broken open and embryo is emerging; Stage 2 = rhizoids present; Stage 3 = leaf primordium developing; Stage 4 = first true leaf emerges; Stage 5 = root and leaf elongation

Seed germination and seedling development were assessed visually using a dissection (stereo) microscope based on seeds that both did and did not contain embryos. Germination and growth stages were characterized on a scale of 0–5 (Fig. 2) according to Zettler and McInnis (1993), where: 0 = no germination (and/or no embryo present); 1 = production of one or more rhizoids (germination); 2 = rupture of the testa by enlarged embryo; 3 = appearance of promeristem (shoot); 4 = appearance of first leaf; 5 = elongation of first leaf, and root initiation. A two-way ANOVA and Tukey-Kramer post hoc analysis were generated using Google Colab, connected to Python 3 in Google Compute Engine backend.

## Results

### Mycorrhizal fungus Pleu1.1

Molecular identification using the BLAST algorithm to identify sequences with the highest level of similarity confirmed the identity of fungus Pleuc1.1 as a *Ceratobasidium* species. The top blast hits included multiple Ceratobasidiaceae species isolates with 100% sequence identity to a partial (77%) overlap, supporting its lineage. Although we are unable to provide a putative species from the sequencing results, as none of the closely related sequences had species epithets, these similar sequences were notable for being from fungal isolates associated with terrestrial orchids (Ercole et al. 2015). These results also confirmed that the fungus obtained and used in seed germination experiments was a pure culture.

### Seed germination and seedling development

**Fig. 3:**
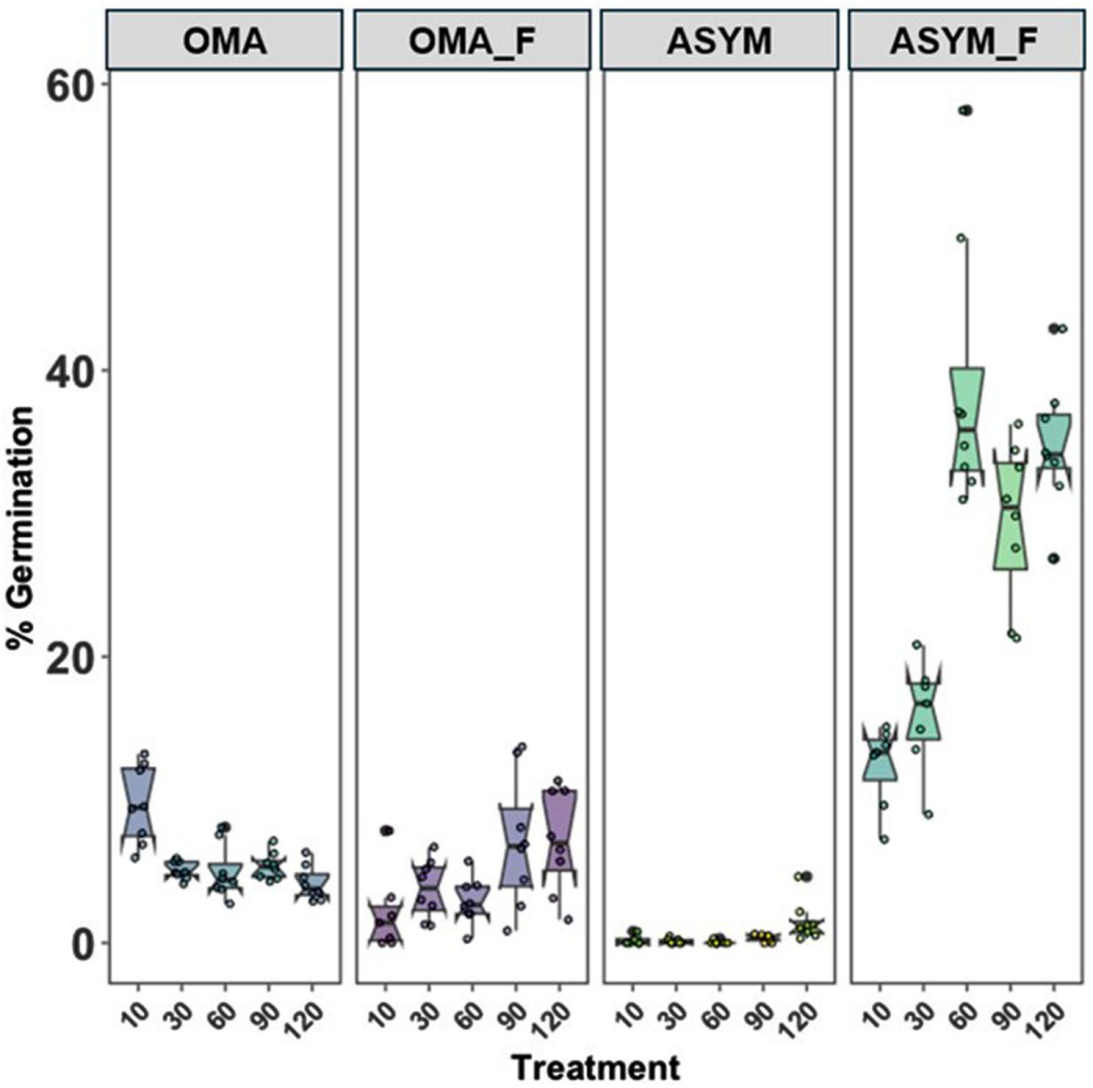
Note that the data collection ended after 385 days post sowing. Each column represents a different treatment (OMA (with Pleuc1.1), OMA_F (with Pleuc1.1 and fluridone) ASYM, and ASYM_F (with fluridone). Within each treatment group are represented the NaOCl (10% household bleach + polysorbate 20 solution) duration times at 10, 30, 60, 90, and 120 min. See Supplementary Materials for the specific data depicted on a table (Supplementary Table 2)

Seed germination (Stage 1) occurred in all symbiotic and asymbiotic treatments, with and without the use of fluridone, 385 days after sowing (Fig. 3). The highest germination percentages were observed on the asymbiotic medium containing fluridone (ASYM_F) at percentages that were at least 2-3X higher than all other treatments (Fig. 3).

Seedling development within some of the replicate plates progressed to Stage 3 in all treatments, but development was marked in all asymbiotic plates containing fluridone (Fig. 3). This was evident in the total number of seeds that developed into seedlings, and by their development to the highest growth stage possible (Stage 5). Thus, only advanced stage seedlings with evidence of leaf elongation were obtained on the asymbiotic medium containing fluridone (Fig. 3).

During data collection, Pleuc1.1 was noted to apparently be completely infecting to the point of overwhelming protocorms and ungerminated seeds in the symbiotic treatment groups. Some protocorms had reached Stage 3, but further growth was inhibited and protocorms appeared to lose their ovoid shape eventually collapsing in the presence of the fungus. As noted in Fig. 3, all of the asymbiotic treatments with fluridone resulted in significantly higher germination percentages than any other treatment group, with the 60-minute bleach treatment reaching the highest germination at 36.32%. In contrast, germination percentages for the asymbiotic groups sans fluridone were below 2% for the asymbiotic groups sans fluridone and were below 10% for all of the symbiotic groups. A two-way ANOVA test found significance in the media type and bleaching time relationship (*p* = 1.7086e-28, see Supplementary Table 1). A Tukey-Kramer post hoc analysis further detailed that asymbiotic groups with fluridone performed significantly better than other groups (*p* < 0.05). Mature seeds of *Platanthera leucophaea* germinated and developed to the protocorm stage in both symbiotic and asymbiotic treatments after incubation for ca. 1 year (385 days) in vitro.

## Discussion

This study confirms that fluridone, an ABA inhibitor, may be used as an effective asymbiotic media additive to stimulate seed germination of a rare temperate terrestrial orchid, *Platanthera leucophaea*, to the leaf-bearing stage in vitro. Our findings, therefore, agree with two reports by Lee et al. (2007, 2015) who experimented with this organic compound to facilitate seed germination and development of two terrestrial orchids native to Asia, *Calanthe tricarinata* and *Cypripedium formosanum*. As a commercial herbicide that leads to chlorophyll degradation, fluridone’s use as a media additive to propagate a photosynthetic orchid seems counterintuitive. However, this compound’s half-life is reported to be ca. three weeks in soil (Magnone et al. 2013), implying that its lingering effects in agar media would not be expected to persist by the time seedlings initiated leaves in vitro (in this case, 385 days). Fluridone also degrades quickly upon exposure to sunlight (Magnone et al. 2013), which may suggest that additional traces of this compound will not persist long in tissues once seedlings are illuminated in a laboratory setting and taken off of media containing fluridone. However, in vitro conditions differ from field conditions, thus fluridone degradation within an in vitro environment needs further study.

The potential for inducing herbicide resistance is a major concern that cannot be ignored when using herbicides within a plant production pipeline, particularly when resulting plants will be going into fragile, natural ecosystems in which those same herbicides may be of preferred use (Schütte et al. 2017). To date, only 2 species are reported to be resistant to fluridone worldwide: *Hydrilla verticillata* and *Raphanus raphanistrum *(Michel et al. 2004; Lu et al. 2020). In both cases, the resistance developed in field conditions under which chronic long-term (i.e., decades) application of PDS-inhibiting herbicides played a key part in the development of resistance. Although we terminated the experiment after fluridone-exposed seedlings were incubated solely in the dark (385 days post-sowing), these seedlings presumably began to initiate photosynthesis approx. 1–2 weeks after being taken off fluridone-containing media, kept in the dark for approx. a month, then exposed to light, as evidenced by their green coloration (unpub. obs.). Mutations within the PDS gene have been implicated in causing target-site resistance to PDS-inhibiting herbicides in *Hydrilla*, but in *Raphanus* the resistance mechanism appears to be related to fast herbicide metabolism via cytochrome P450s (Michel et al. 2004; Lu et al. 2020). Two methods to check for fluridone resistance can be to take sub-samples of seedlings and place them back on fluridone-containing media under the light to observe for loss of pigmentation and decline (total loss of seedlings, but indicative of resistance), or to check for PDS mutations and enhanced herbicide metabolism via cytochrome P450s in individuals prior to outplanting (no loss in seedlings). We note that at present, fluridone is registered for use primarily as an aquatic herbicide with some additional agricultural uses (Herbicide Handbook Committee 2014; Environmental Protection Agency 2023; Butts et al. 2024), and is not labeled for use in areas in which *P. leucophaea* seedlings would be outplanted; however, labeling approval can change in the future. The rarely documented occurrence of resistance does suggest our application of fluridone presents a low risk of inducing resistance, but we stress the need to proceed with precaution until studies investigating potential fluridone resistance per our application can be conducted. The benefits of our protocol might provide future researchers with the means to explore the use of fluridone in germinating *P. leucophaea*, and/or other, hardy difficult-to-germinate terrestrial orchid species.

Because we did not document nor quantify the presence (localization) of ABA in seeds, definitive statements regarding the mechanisms behind our results using fluridone have yet to be confirmed, arguing in support of more study. Nevertheless, previous work by Lee et al. (2007, 2015) allowed us to extrapolate results from the available literature which suggest that ABA’s influence may be weakened by fluridone. We urge other researchers to explore this possibility further for *P. leucophaea* and other temperate terrestrial orchids that have a reputation for being difficult to propagate. Specifically, experiments are needed to confirm the amount of ABA present in the seed, and also to test whether or not fluridone is, in fact, inhibiting ABA by replicating the methods of existing studies (Lee et al. 2015) in *P. leucophaea* and other species of interest. Immunoflorescent localization tests investigating ABA levels in seeds sown symbiotically and asymbiotically at different sterilization times might help to answer these questions, as demonstrated by Lee et al. 2015). Moreover, seed baiting (Rasmussen and Whigham 1993) might be a useful technique to utilize for testing ABA localization in seeds that germinate naturally in situ in the presence of mycorrhizal fungi. Because *P. leucophaea* is a species that responds favorably to periodic burning in its tallgrass prairie habitat (Bell et al. 2021), the role of smoke at breaking ABA-induced seed dormancy may also be explored as Kamran et al. (2013) suggested. Seed baiting may also reveal other environmental ABA biosynthesis inhibitors that might be involved. In the meantime, our study offers a new and potentially significant protocol for stimulating germination as a media additive for propagating this rare hardy species more effectively from seed.

Another surprising finding revealed by our study was the lower seed germination percentages observed when seeds were sown on oat-based media (OMA) and inoculated with a mycorrhizal fungus, including OMA containing fluridone (Fig. 3). Not only were germination percentages lower in the two symbiotic treatments compared to the asymbiotic medium with added fluridone, only a few seedlings developed to Stage 3, and none yielded leaves (Stages 4 and 5) after 385 days of incubation. The fact that some (albeit few) of the embryos developed to the protocorm stage on OMA suggests that our particular *Ceratobasidium* strain did have some intrinsic ability to serve as a mycorrhizal associate, but other *Ceratobasidium* stains should be explored for their symbiotic potential to facilitate development to Stage 5 mirroring previous studies (e.g., Zettler et al. 2001) involving this same orchid species. As is often the case using symbiotic protocols, generally a smaller number of embryos eventually develop to the protocorm stage, even when initial germination percentages are much higher, and we see this same pattern in Table 1. Thus, the presence of fluridone did not lower germination rates, because germination with and without this additive was comparable in both OMA treatments. We speculate that either the fungus has some detrimental effects on germination, or that the fungus may be degrading and/or altering fluridone activity – perhaps a scenario in which the fungus is taking up the fluridone, decreasing the dose available to the seeds (Hao et al. 2010). The fluridone may have additional impacts on fungal metabolite production that could differently impact seedling development (Hao et al. 2010); however, this needs further study.

Fungi in the genus *Ceratobasidium* have been isolated with regularity throughout the species’ range, and from all growth stages (protocorms, seedlings, mature plants) in studies that date back to Zettler et al. (2001) and even earlier (e.g., Curtis 1939). A recent report by Thixton et al. (2020) provided a list of various *Ceratobasidium* strains isolated from *P. leucophaea* throughout its natural range over a span of a decade, augmenting earlier literature (e.g., Zettler and Piskin 2011). Some of these *Ceratobasidium* strains appear to vary genetically based on molecular evidence using ITS amplification (e.g., Thixton 2017). The fact that our strain of *Ceratobasidium* used in this study facilitated development to the protocorm stage suggests this fungus is a mycorrhizal associate, but its use did not result in higher growth stages (leaf development) unlike some other *Ceratobasidium* strains used in earlier symbiotic germination experiments e.g., (Zettler et al. 2001, 2005). While the fungus utilized in our study was confirmed as a *Ceratobasidium* species (Pleuc 1.1) (Thixton et al. 2020), it may represent a strain that had little to do with facilitating seed germination at the time it was collected given it was acquired from a mature plant. This is plausible considering that orchid roots often become colonized by a mixture of fungi as they mature, many of which may be of little physiological significance to the orchid (Meng et al. 2019). Rasmussen (1995) reported that fungal hyphae are capable of entering orchid tissues through living rhizoids on roots, rhizomes and protocorms, and even through epidermal cells and secondary root tissues (e.g., dead velamen cells). Zelmer, Cuthbertson and Currah (1997) reported isolating fungi from different genera in various growth stages. Several researchers (e.g., Milligan and Williams 1988; Sharma 2002; Sharma et al. 2003) reported that fungi in younger orchid stages were different from those obtained from adult stages, and that the fungi from the latter were less effective at germinating seeds compared to fungal isolates obtained from younger stages (e.g., protocorms), lending support for the concept of ‘fungal succession’. Thus, this particular strain may lack the intrinsic ability to facilitate seed germination which would explain why the fungus was able to germinate a small percentage of the seeds, and why the protocorms did not survive at the conclusion of the study. Previous studies that utilized different strains of *Ceratobasidium* (Zettler et al. 2001, 2005; Bowles et al. 2002) reported more favorable outcomes (e.g., higher germination percentages leading to leaf-bearing seedlings), suggesting that the symbiotic technique should not be discounted in light of our success using fluridone on asymbiotic media.

While symbiotic seed germination has often shown considerable promise for growing terrestrial orchids from seed, *P. leucophaea* has been difficult to propagate symbiotically leading to seedling reintroduction with standard protocols, and many specialists have resorted to using asymbiotic techniques instead as with the case of *Platanthera* (*Peristylus*) *holochila* in Hawaii, for example (see Zettler et al. 2011) Our method using fluridone represents one ‘tool’ in the conservation ‘tool kit’ for recovery by providing a rapid technique for germinating many more embryos leading to high (leaf-bearing) growth stages than previous techniques have reported. We do not suggest the use of this herbicide when a suitable fungus has been identified for the use of seedlings to be returned to the general location range from where the fungus has been isolated, but rather to use fluridone to produce asymbiotically-grown seedlings for the purpose of repopulating extinct populations or establishing new populations to prevent the introduction of a foreign fungus to those soils. Furthermore, it remains to be determined if, and to what extent, the seedlings obtained using this herbicide would be able to survive once deflasked. As Anderson (1991) demonstrated, for example, seedlings of *Spiranthes magnicamporum* grown with mycorrhizal fungi had superior survival outcomes *ex vitro* compared to seedlings grown on asymbiotic media, suggesting that roots that harbor fungi benefit the acclimatization process. Until more effective techniques are developed in the coming years that improve germination, development and establishment of this and other temperate terrestrial orchids, the use of fluridone in asymbiotic germination may have a practical use in conservation and/or horticultural purposes.

## Conclusions

Our study demonstrates that fluridone added to asymbiotic media P723 (PhytoTechnology Labs) prior to sowing stimulates in vitro seed germination of *Platanthera leucophaea* leading to leaf-bearing seedlings obtained in a little over one year (385 days). Previous work by Lee et al. (2007, 2015) allowed us to extrapolate results from the available literature which suggest that ABA’s influence on inhibiting seed germination may be weakened artificially by fluridone, a commercial herbicide. Because we did not document nor quantify the presence (localization) of ABA in seeds, definitive statements regarding the mechanisms behind our results using fluridone have yet to be confirmed, arguing in support of more study. Nevertheless, this new technique may have merit for propagating this and other hardy orchid species for conservation and/or horticultural purposes.

## Supplementary Information

Below is the link to the electronic supplementary material.

Supplementary Materials

## Data Availability

The authors confirm that local and national guidelines and legislation are followed by acquiring appropriate permissions and licenses for the study.
