# Supplementary material for: Fluridone stimulates in vitro seed germination of a rare hardy terrestrial orchid (Platanthera leucophaea)
Source: Bot Stud. 2025 Oct 30;66:37. doi: 10.1186/s40529-025-00484-w (PMC12575895; doi:10.1186/s40529-025-00484-w)
Supplement: Supplementary file 1 — Supplementary Materials [file 40529_2025_484_MOESM1_ESM.docx]

**SUPPLEMENTARY MATERIALS**

**Supplementary Table 1:** Two-way ANOVA table comparing effects of treatment media (OMA, OMA_F, ASYM, ASYM_F) and sterilization time (NaOCL + polysorbate 20 solution at 10, 30, 60, 90, and 120 minutes).

|  | **Sum_sq** | **df** | **F** | **PR(>F)** |
| --- | --- | --- | --- | --- |
| **Media** | **16094.6** | **3** | **460.064** | **2.60909e-71** |
| **Time** | **947.213** | **4** | **20.3071** | **3.74236e-13** |
| **Media : Time** | **3436.59** | **12** | **24.5587** | **1.70863e-28** |
| **Residual** | **1597.57** | **137** | **nan** | **nan** |

| **Supplementary Table 2:** A comparison of in vitro symbiotic vs asymbiotic germination of *Platanthera leucophaea* by growth stage 385 days after sowing across multiple treatments that tested bleach exposure times with and without fluridone. | | | | | | | | | | | |  |
| --- | --- | --- | --- | --- | --- | --- | --- | --- | --- | --- | --- | --- |
| **Treatment** | **Symbiont** | **n*** | **Scar Time** | **# Seeds** | **Stage 0** | **Stage 1** | **Stage 2** | **Stage 3** | **Stage 4** | **Stage 5** | **% Germination** |  |
| **ASYM** | - | 8 | 10 | 3568 | 3562 | 0 | 3 | 3 | 0 | 0 | 0.17 |  |
| **ASYM** | - | 8 | 30 | 3476 | 3472 | 0 | 2 | 1 | 1 | 0 | 0.12 |  |
| **ASYM** | - | 8 | 60 | 3034 | 3033 | 0 | 0 | 0 | 1 | 0 | 0.03 |  |
| **ASYM** | - | 8 | 90 | 3554 | 3541 | 7 | 2 | 3 | 1 | 0 | 0.37 |  |
| **ASYM** | - | 8 | 120 | 4108 | 4051 | 46 | 4 | 7 | 0 | 0 | 1.39 |  |
| **ASYM_F** | - | 7 | 10 | 2599 | 2284 | 39 | 37 | 80 | 158 | 1 | 12.12 |  |
| **ASYM_F** | - | 7 | 30 | 2376 | 2004 | 63 | 38 | 54 | 217 | 0 | 15.66 |  |
| **ASYM_F** | - | 8 | 60 | 2274 | 1448 | 132 | 59 | 114 | 517 | 4 | 36.32 |  |
| **ASYM_F** | - | 8 | 90 | 2820 | 1997 | 149 | 51 | 111 | 503 | 9 | 29.18 |  |
| **ASYM_F** | - | 8 | 120 | 2642 | 1737 | 180 | 58 | 83 | 579 | 5 | 34.25 |  |
| **OMA** | Pleuc1.1 | 8 | 10 | 3486 | 3148 | 322 | 12 | 4 | 0 | 0 | 9.7 |  |
| **OMA** | Pleuc1.1 | 8 | 30 | 3420 | 3247 | 158 | 14 | 1 | 0 | 0 | 5.06 |  |
| **OMA** | Pleuc1.1 | 8 | 60 | 3732 | 3547 | 182 | 3 | 0 | 0 | 0 | 4.96 |  |
| **OMA** | Pleuc1.1 | 8 | 90 | 3436 | 3252 | 181 | 3 | 0 | 0 | 0 | 5.36 |  |
| **OMA** | Pleuc1.1 | 8 | 120 | 3538 | 3394 | 144 | 0 | 0 | 0 | 0 | 4.07 |  |
| **OMA_F** | Pleuc1.1 | 7 | 10 | 2840 | 2785 | 39 | 16 | 0 | 0 | 0 | 1.94 |  |
| **OMA_F** | Pleuc1.1 | 8 | 30 | 3039 | 2926 | 95 | 18 | 0 | 0 | 0 | 3.72 |  |
| **OMA_F** | Pleuc1.1 | 8 | 60 | 3046 | 2955 | 72 | 18 | 1 | 0 | 0 | 2.99 |  |
| **OMA_F** | Pleuc1.1 | 8 | 90 | 3169 | 2943 | 187 | 39 | 0 | 0 | 0 | 7.13 |  |
| **OMA_F** | Pleuc1.1 | 8 | 120 | 3017 | 2795 | 150 | 72 | 0 | 0 | 0 | 7.36 |  |
| Note that all ASYM_F groups out-performed other treatments, with the best germination percents occurring when seeds were exposed to NaOCl (10% household bleach + polysorbate20 solution) for durations of 60-120 minutes. | | | | | | | | | | | |  |
|  |  |  |  |  |  |  |  |  |  |  |  |  |
|  |  |  |  |  |  |  |  |  |  |  |  |  |
| ***Number of replicate petri plates (n) for a given treatment. Contaminated plates were discarded, accounting for unequal sample sizes.** | | | | | | | | | | | |  |
